# Increased microglia activation in late non‐central nervous system cancer survivors links to chronic systemic symptomatology

**DOI:** 10.1002/hbm.26491

**Published:** 2023-09-26

**Authors:** Poppy L. A. Schoenberg, Alexander K. Song, Emily M. Mohr, Baxter P. Rogers, Todd E. Peterson, Barbara A. Murphy

**Affiliations:** ^1^ Department of Physical Medicine and Rehabilitation Vanderbilt University Medical Center Nashville Tennessee USA; ^2^ Osher Center for Integrative Health Vanderbilt University Medical Center Nashville Tennessee USA; ^3^ Department of Neurology Vanderbilt University Medical Center Nashville Tennessee USA; ^4^ Vanderbilt Brain Institute Vanderbilt University Nashville Tennessee USA; ^5^ Department of Radiology and Radiological Sciences Vanderbilt University Medical Center Nashville Tennessee USA; ^6^ Division of Hematology and Oncology Vanderbilt‐Ingram Cancer Center Nashville Tennessee USA

**Keywords:** cancer survivorship, diffusion tensor imaging, head and neck cancer, microglial activation, neuroinflammation, non‐CNS cancer, positron emission tomography, systemic symptomatology

## Abstract

Prolonged inflammatory expression within the central nervous system (CNS) is recognized by the brain as a molecular signal of “sickness”, that has knock‐on effects to the blood–brain barrier, brain‐spinal barrier, blood‐cerebrospinal fluid barrier, neuro‐axonal structures, neurotransmitter activity, synaptic plasticity, neuroendocrine function, and resultant systemic symptomatology. It is concurred that the inflammatory process associated with cancer and cancer treatments underline systemic symptoms present in a large portion of survivors, although this concept is largely theoretical from disparate and indirect evidence and/or clinical anecdotal reports. We conducted a proof‐of‐concept study to link for the first time late non‐CNS cancer survivors presenting chronic systemic symptoms and the presence of centralized inflammation, or neuroinflammation, using TSPO‐binding PET tracer [^11^C]‐PBR28 to visualize microglial activation. We compared PBR28 SUVR in 10 non‐CNS cancer survivors and 10 matched healthy controls. Our data revealed (1) microglial activation was significantly higher in caudate, temporal, and occipital regions in late non‐central nervous system/CNS cancer survivors compared to healthy controls; (2) increased neuroinflammation in cancer survivors was not accompanied by significant differences in plasma cytokine markers of peripheral inflammation; (3) increased neuroinflammation was not accompanied by reduced fractional anisotropy, suggesting intact white matter microstructural integrity, a marker of neurovascular fiber tract organization; and (4) the presentation of chronic systemic symptoms in cancer survivors was significantly connected with microglial activation. We present the first data empirically supporting the concept of a peripheral‐to‐centralized inflammatory response in non‐CNS cancer survivors, specifically those previously afflicted with head and neck cancer. Following resolution of the initial peripheral inflammation from the cancer/its treatments, in some cases damage/toxification to the central nervous system occurs, ensuing chronic systemic symptoms.

## Graphical abstract

Here we present data that links for the first time the presence of microglial activation (or neuroinflammation) with the presentation of chronic systemic symptoms in non‐central nervous system cancer survivors.

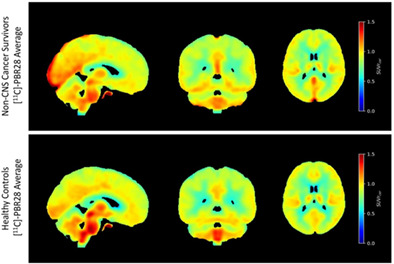

## 1 INTRODUCTION

Non‐central nervous system (CNS) cancers entail profound inflammatory signaling within the central nervous system (i.e. neuroinflammation), that when prolonged cause centralized toxicity. Multiple inflammatory mediators, including cytokines/chemokines, prostaglandins, proteases, and Danger‐Associated‐Molecular‐Patterns (or DAMPs), participate in cellular ‘communication’ within the tumor microenvironment and its consequential systemic response to tumor presence/status (Candido & Hagemann, 2013; Mantovani et al., 2010). Cancer treatments also activate the immune system and proinflammatory cytokine production via Protection‐Associated‐Molecular‐Patterns' (PAMPs), such as release of neutrophil/monocyte, cytokine interleukins, and tumor necrosis factor (TNF) facilitated by glia cells during early innate immune response (Mϋller, 2017; Tabas & Glass, 2013). Radiation and/or chemoradiation treatments have received the most attention for their ability to induce significant proinflammatory response, albeit even newer treatments involving targeted therapy, hormone therapy, and immunotherapy, also appear to cause toxicity within the central nervous system in non‐CNS cancer survivors (Kroschinsky et al., 2017; Stone & DeAngelis, 2016). Despite peripheral and central neuroimmune‐inflammatory contributions to central nervous system toxicity, extant clinical focus pertains to peripheral cytokine markers (Cleeland et al., 2003), particularly since the initial insult for non‐CNS cancers activate peripheral inflammatory responders and mediators. However, the multicellular dynamics of this inflammatory process are mediated by non‐neuronal cells of the central nervous system, including astrocytes and the brain's resident macrophages, microglia. As such, microglia might provide a much needed centralized marker of neuroinflammation in non‐CNS cancer survivors to gauge injury to the central nervous system in patients. Peripheral injury/trauma/cancer release inflammatory mediators that activate glial components of peripheral and central cellular circuitry (Diakos et al., 2014; Jha et al., 2014). Prolonged inflammatory expression within the CNS is recognized by the brain as a molecular signal of “sickness”, that has knock‐on effects to the blood–brain barrier, brain–spinal barrier, blood–cerebrospinal fluid barrier, neuro‐axonal structures, neurotransmitter activity, synaptic plasticity, neuroendocrine function, and resultant symptomatology (O'Reilly & Tom, 2020a; Raison et al., 2006).

Chronic systemic symptoms often present in survivors well after cancer outcomes have improved and significantly reduce quality of life, including physical limitations, cognitive impairment, mood dysregulation, sleep problems, fatigue, centralized pain, sexual dysfunction, and hypothalamic abnormalities manifested as thermal discomfort or hyperhidrosis (Harrington, Hanse, et al., 2010; Horng‐Shiuann & Harden, 2015; Murphy et al., 2007). Oncologists concur that the inflammatory process associated with cancer and cancer treatments underline systemic symptoms present in a large portion of survivors, although this concept is largely theoretical from disparate and indirect evidence and/or clinical anecdotal reports. Extant research focuses on animal models, neurovascular measures such as MRI, and peripheral markers of inflammation (Andryszak et al., 2017; Bower et al., 2007; Collado‐Hidalgo et al., 2006; Santos & Pyter, 2018; Seigers et al., 2009, 2010; Yang et al., 2011), opposed to a direct marker of neuroinflammation in humans. Until now, no central molecular marker of inflammation has been linked to systemic symptoms in survivors of non‐CNS cancers. Here, we report a proof‐of‐concept study to examine the role of microglial activation in non‐CNS survivors (specifically head and neck cancers since this was the clinical cohort available for recruitment) who present systemic symptoms for at least 6‐months post remission.

Microglia are critical nervous system‐specific neuroimmune cells that serve as tissue‐resident macrophage and have unique immunological properties through the ability to shift function based on a process of polarization (Orihuela et al., 2016). In their non‐pathological—or homeostatic—state they regulate neuronal activity and synaptic plasticity. Brain homeostasis is in part attributed to the role of microglia to regulate inflammation, cytotoxicity, repair, and regeneration of neural cells (Dupont et al., 2017). However, in their pathological—or activated—state (the DAMP–PAMP process), they serve critical neuro‐immunological functions by recruiting large scale pro‐inflammatory cytokine and peripheral immune cell production towards pathogen destruction, debris clearance, and tissue repair (Woodburn et al., 2021). In this activated state microglia upregulate the expression of the benzodiazepine receptor (Papadopoulos et al., 2006), renamed as the translocator protein 18 kDa (TSPO) to better characterize the subcellular roles and molecular functions of this protein (Best et al., 2019; Papadopoulos et al., 2006). TSPO is a highly hydrophobic five transmembrane domain protein expressed in the outer mitochondrial membrane of microglia and other cells of macrophage lineage (Papadopoulos et al., 2006), and is significantly upregulated in microglial cells in patients with neurological degeneration such as Alzheimer's disease, Parkinson's disease, Huntington's disease, and multiple sclerosis (Dupont et al., 2017; Gómez‐Nicola et al., 2013; Lull & Block, 2010; Yin et al., 2017). Overexpression of TSPO in microglia is also acutely abundant in the core infarction of focal cerebral ischemia and following traumatic brain injury/stroke (Dupont et al., 2017; Hernandez‐Ontiveros et al., 2013; Jiang et al., 2020; Li et al., 2021). In sum, microglial activation represents a key pathological mechanism associated with injury, neuronal dysfunction, neurodegeneration, and disease progression. TSPO expression in microglial cells as a molecular marker of neuroinflammation can be visualized using Positron Emission Tomography (PET) imaging. Here, we specifically used the PET tracer [^11^C]PBR28, a second generation TSPO radioligand with 80‐fold higher specific binding for activated brain glia compared with the first generation [^11^C]PK11195, the prototypical TSPO radioligand used widely to assess TSPO PET (Kreisl et al., 2010).

In order to collect a multi‐dimensional dataset to inform mechanistic interpretation, our methodology also included Diffusion tensor imaging/DTI. DTI visualizes intra‐voxel tissue microarchitecture towards the non‐invasive assessment of white matter microstructural integrity, reconstruction of major fiber bundles, and levels of cerebrospinal fluid. Axon‐myelin units of the fiber tracts cause anisotropic water diffusion that subsequently constitute the DTI signal. We calculated fractional anisotropy as a metric of the directional coherence of diffusion along white matter fibers, where 0 indicates no ordered pattern (isotropy) and 1 indicates locally uniform fiber tract pattern (anisotropy) (Basser & Pierpaoli, 1996). Lower fractional anisotropy indicates reduced brain tissue microstructural integrity, or fiber tract organization, representing a marker of neurological deterioration (Pasternak et al., 2015). In sum, we hypothesized that compared to matched healthy controls, cancer survivors would yield higher microglial activation measured by TSPO expression, and lower fractional anisotropy measured by DTI. To be able to distinguish between centralized versus peripheral inflammation, we also collected blood serum cytokine and chemokine markers to provide information of the latter. Finally, we collected clinical measures of systemic symptomatology and neurocognitive function to investigate the interaction between our neuroimaging data and clinical outcomes. The aim was to collect data to support our proof‐of‐concept that non‐CNS cancer survivors who present systemic symptomatology have continued centralized toxicity and inflammation of the CNS, that is, neuroinflammation.

## 2 METHODS

### 2.1 Design and sample

This study was conducted at the Vanderbilt University Institute of Imaging Science. Tables 1 and 2 present demographics and clinical measures of the study sample. A retrospective cohort study design compared 20 participants; 10 head and neck cancer survivors who were cancer free and had a mean 4.6 years (range 1–16) from their final successful treatment, versus 10 healthy age‐, sex‐, and education‐matched control participants. Eligibility criteria for cancer survivors were: (1) age ≥ 21, (2) previous head and neck cancer of larynx, pharynx, oral cavity paranasal sinus, salivary gland, or unknown primary, (3) any histology of any epithelial origin, (4) completed therapy a minimum of 6 months prior to study entry, (5) at least two systemic symptoms on the general symptom subscale of the Vanderbilt Head and Neck Symptom Survey, (6) no history of neurodegenerative disease, unrelated to cancer history/treatment, (7) English language ability to understand instructions and be able to provide informed consent. Exclusion criteria for all study participants (survivors and healthy controls) were: (1) alcohol/substance abuse/dependence within the last 6 months, (2) current or previous co‐morbid bipolar disorder, psychosis, obsessive compulsive disorder, eating disorders, personality disorders, (3) neurological disorders (unrelated to cancer/treatment for survivors), for example, ADHD, ASDs, epilepsy, (4) learning difficulties impeding comprehension for providing consent and/or during experimentation. Cancer survivors were recruited from the Vanderbilt Department of Otolaryngology, Vanderbilt Head and Neck Cancer clinics. Matched healthy controls were recruited via VUMC recruitment listserv and ResearchMatch. All research procedures were in accordance with the Vanderbilt University Institutional Review Board and the Vanderbilt‐Ingram Cancer Center's Scientific Review Committee approval.

**TABLE 1:** Demographic data.

	Cancer survivors	Healthy controls	Comparison
Sex; *N* (%)			*χ* ^2^(1) = 1.49, *p* = .223
Man	9 (90%)	7 (70%)	
Woman	1 (10%)	3 (30%)	
Mean age; *M* (SD)	63 (6.48)	63 (8.38)	*t*(19) = −0.248, *p* = .807
Racial classification; *N* (%)			*χ* ^2^(2) = 1.18, *p* = .555
American Indian/Alaskan Native	1 (10%)	1 (10%)	
Asian	0 (0%)	0 (0%)	
Native Hawaiian or other Pacific Islander	0 (0%)	0 (0%)	
Black or African American	0 (0%)	1 (10%)	
White	9 (90%)	8 (80%)	
Education; *M* (SD)	13.80 year (4.13)	16.60 year (2.07)	*t*(15.02) = −1.72, *p* = .105
Medication; *N*			
Analgesic/opioid	5		
Anesthetic	1		
Narcotic	1		
NSAID	2		
Sedative	4		
SSRI	3		
Type of cancer			
Oropharynx	9 (10%)		
Nasopharynx	1 (10%)		
Nasal cavity	0 (0%)		
Larynx	0 (0%)		
Total treatment received			
Surgery + ChemoXRT	1 (10%)		
Induction, surgery, ChemoXRT	3 (30%)		
Induction + ChemoXRT	2 (20%)		
ChemoXRT	3 (30%)		
Radiotherapy only	1 (10%)		
Length of radiation treatment (months); *M* (SD)	1.52 (0.28)		
Number of induction chemotherapy cycles; *M* (SD)	6.6 (2.30)		
Number of ChemoXRT cycles; *M* (SD)	7.22 (1.09)		
Tumor staging PRIOR to disease‐free status			
Cancer stage T (original tumor size)	1.75 (.463)		
Cancer stage N (reginal lymph node involvement)	1.78 (.667)		
Cancer stage^a^	2.40 (1.342)		

^a^ 0 = 0; 1 = I; 2 = II; 3 = III; 4 = IVa; 5 = IVb; 6 = IVc.

**TABLE 2:** Clinical outcomes.

Clinical measure *M* (SD)	Cancer survivors	Healthy controls	Comparison
VHNSS	163.8 (61.31)	10.2 (10.20)	***p* < .001**
GSS	48 (23.29)	5.7 (5.70)	***p* < .001**
PROMIS‐29			
Anxiety	7.5 (3.95)	4.5 (0.71)	***p* = .047**
Depression	8.1 (3.76)	4.3 (0.68)	***p* = .003**
Fatigue	13.4 (3.41)	5.2 (1.48)	***p* < .001**
Sleep disturbance	12.1 (4.12)	6.6 (1.51)	***p* = .001**
Social	12.7 (5.23)	4.3 (1.06)	***p* < .001**
Pain	9.9 (5.71)	4.4 (1.35)	***p* = .006**
Neurotoxicity	37.8 (24.71)	4.4 (4.35)	***p* < .001**
Quality of life	38.3 (11.95)	56.1 (2.89)	***p* < .001**
BRIEF			
Inhibit	5.82 (1.52)	4.9 (0.88)	*p* = .118
Shift	10.18 (2.41)	6.6 (0.84)	***p* < .001**
Emotional control	15.73 (3.88)	11.5 (2.32)	***p* = .004**
Initiate	14.55 (4.22)	8.9 (1.37)	***p* < .001**
Working memory	14.36 (3.47)	7.5 (0.71)	***p* < .001**
Plan/organize	17.82 (4.84)	11.3 (2.45)	***p* < .001**
Organization of materials	13.27 (3.09)	10.4 (3.84)	*p* = .097
Task monitor	10.90 (1.52)	6.6 (0.97)	***p* < .001**
Self‐monitor	10.18 (1.70)	6.9 (1.60)	***p* < .001**

*Note*: Vanderbilt Head and Neck Symptom Survey Version 2.0 (VHNSS), VHNSS General Symptom Survey (GSS). Values in bold font highlights statistically significant findings.

### 2.2 Procedure

The study was approved by the Vanderbilt Institutional Review Board (IRB), and informed written consent was obtained prior to experimental testing. Potential cancer survivor patients were initially identified by a medical oncologist and followed up by the research team for specific research‐based screening. Disease and treatment histories were reviewed from medical records to reduce burden on patients. Potential healthy control participants were provided a link to HIPAA‐compliant RedCap screening surveys to ascertain eligibility criteria. Participants completed an MRI scan, separate CT‐PET scan, provided a blood sample, and completed self‐report surveys. At the end of the study, all participants received financial compensation as a token of gratitude for their time.

### 2.3 MRI and PET image acquisition

All MRI scans were acquired to provide high resolution structural delineation for quantification of [^11^C]‐PBR28‐TSPO standard uptake value ratio (SUVR). MRIs were collected with a 3.0 T Philips (Philips Intera Achieva, Philips Healthcare) imaging scanner using body coil transmission and 32‐channel SENSE array reception. Structural (T1‐weighted MPRAGE, FOV = 256 × 256 mm, 1 mm isotropic resolution, TE = 2 ms, TR = 8.95 ms and TI = 643 ms; 5‐min) and high angular resolution diffusion‐weighted imaging/DWI (HARDI) scans (2.5 mm isotropic resolution, multi‐band acquisition, FOV = 96 × 96 mm, TR = 2.65 s, TE = 101 ms, Gmax = 37.5 mT/m), two shells, 1000 s/mm^2^ (24 directions) and 2000 s/mm^2^ (60 directions), evenly distributed on the sphere that support reverse phase encoding for distortion correction; 8‐min), were acquired to better characterize anatomical features of the brain. For eddy current correction, the diffusion‐weighted imaging was affinely registered to *b*0 with 12 degrees of freedom using FLIRT in FSL 5.0. The registration matrix of each diffusion‐weighted image was then be used to measure patient movement and gradient table rotated accordingly.

All PET scans were acquired on a Philips Vereos PET/CT scanner with a 3D emission acquisition and a transmission attenuation correction. [^11^C]‐PBR28 was synthesized according to standard procedures by the VUIIS Radiochemistry Core. Participants underwent an intravenous injection of 555 MBq (average 15.465 mCi, range 13.989–16.196 mCi) as a slow bolus over a 30‐s period, immediately followed by flushing of the catheter with a 0.9% saline solution. PET scanning commenced ~50 min after injection, and emission data was acquired for a total of 40 min (~50–90 min post injection). A low‐dose CT image was acquired immediately prior to PET imaging for use in scatter and attenuation corrections and to facilitate registration of PET data to MR images. The listmode PET data was reconstructed using the scanner's OSEM‐based algorithm (3 iterations, 21 subsets) into a series of eight images of 5‐min duration each. Each image was reconstructed on a 128 × 128 × 62 grid of 2‐mm isotropic voxels. Recording of patient weight and injected activity in the scanner software allowed images to be displayed in standardized uptake value (SUV).

### 2.4 Diffusion tensor imaging/DTI analysis

Diffusion imaging processing and quality assurance was performed with the PreQual pipeline (Cai et al., 2021). Each patient's structural scan was segmented using spatially localized atlas network tiles (SLANT) method (Huo et al., 2018, 2019; Xiong et al., 2019), able to segment a 3D MRI brain scan into 132 anatomical regions. Individual T1 MR images were segmented and GM surfaces derived using MaCRUISE, reconstructing inner, central, and outer cortical surfaces via the topology‐preserving geometric deformable surface model. GM central surface was normalized to the MNI space by applying inverse deformation field to the vertices. *Diffusion data analysis*: The diffusion data were preprocessed and quality‐checked with the following pipeline built around the MRTrix3 (Tournier et al., 2019), FSL (Jenkinson et al., 2012), and ANTs (Tustison et al., 2014) software packages. First, any volumes with a corresponding *b* value less than 50 were treated as *b*0 volumes for the remainder of the pipeline. Then, the diffusion data were denoised with the provided dwi‐denoise function included with MRTrix3 (Cordero‐Grande et al., 2019; Veraart, Fieremans, & Novikov, 2016; Veraart, Novikov, et al., 2016). The images were then intensity‐normalized to the first image and concatenated for further processing. No reverse phase encoded images were acquired, but corresponding T1 images of the subjects were available. Thus, a T1 image was used to generate a synthetic susceptibility‐corrected *b*0 volume using SYNB0‐DISCO, a deep learning framework by Schilling et al. (2019). This synthetic *b*0 image was used in conjunction with FSL's topup to correct for susceptibility‐induced artifacts in the diffusion data. FSL's eddy algorithm was then used to correct for motion artifacts and eddy currents and to remove outlier slices (Andersson et al., 2003, 2016; Andersson & Sotiropoulos, 2016; Smith et al., 2004). Lastly, the preprocessed data were fitted with a tensor model using the dwi2tensor function included with MRTrix3 using an iterative reweighted least squares estimator (Veraart et al., 2013). The tensor fit was converted to a fractional anisotropy (FA) image (Basser et al., 1994; Westin, 1997). The ICBM FA MNI atlas with 48 white matter tract labels provided with FSL were then non‐rigidly registered to the subject's FA image with the ANTs software package (Avants et al., 2008; Hua et al., 2008; Mori et al., 2005; Wakana et al., 2007). The average FA for each tract was then quantified.

### 2.5 MRI cortical thickness analysis

Since cortical thinning has been shown to be a marker of neurodegeneration (Young et al., 2020), we also conducted posthoc analyses of cortical thickness measures ascertained from the collected MRI T1‐weighted images. Cortical reconstruction and volumetric segmentation were performed with the Freesurfer image analysis suite version 7.2.0, which is documented and freely available for download online (http://surfer.nmr.mgh.harvard.edu/). The technical details of these procedures are described in prior publications (Dale et al., 1999; Desikan et al., 2006; Fischl et al., 1999, 2004; Fischl & Dale, 2000).

### 2.6 PET data analysis

**FIGURE 1:**
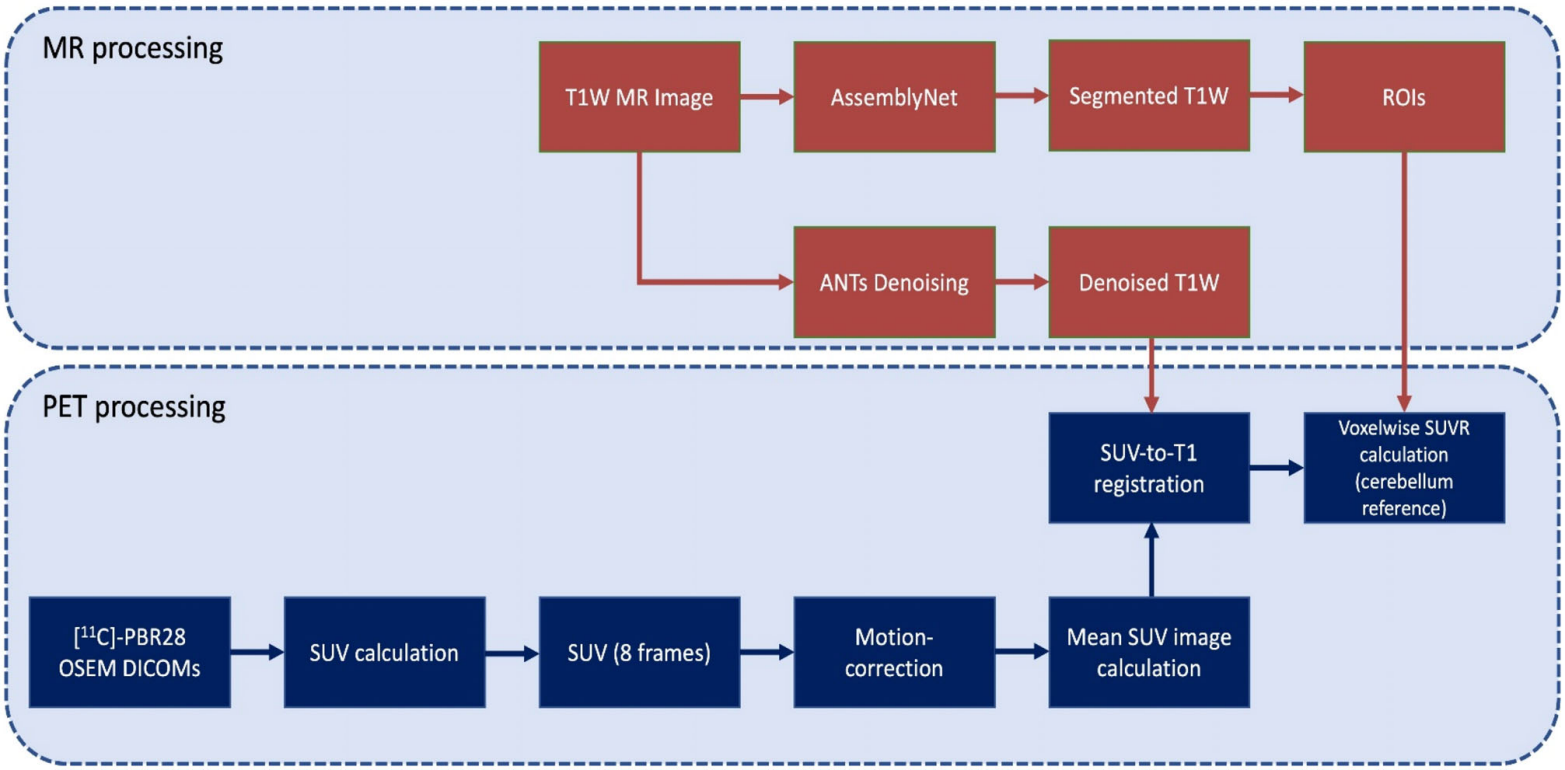
[^11^C]‐PBR28 PET SUVR processing pipeline. Participants' PET imaging data are reconstructed using an ordered subset expectation maximization (OSEM) algorithm. Standardized uptake value (SUV) maps at each time point (8 frames between 50 and 90 min) are calculated based on time of acquisition post‐injection, injection dose, and participant weight. After motion‐correction, a mean SUV image is calculated across the 8 time points. Participants' T1W MRI data are denoised using the nonlocal means filter in the ANTs neuroimaging toolkit, and segmented using AssemblyNet. Participants' mean SUV images are then registered to corresponding T1W MRI space using FSL's FLIRT. Participants' SUVR maps are then calculated by voxelwise division by the whole cerebellum SUV.

Figure 1 provides a schematic overview of the PET data processing pipeline. Data were first be reconstructed into shorter images (<5‐min) to facilitate any required realignment to correct for motion. After the images were motion‐corrected, SUV images were calculated based on injection activity and patient weight. SUV images were then registered to the patient's corresponding MR image using ANTs rigid transformation algorithm (Avants et al., 2011). Participant‐specific MR images were used for region‐of‐interest (ROI) segmentation using the AssemblyNet segmentation framework (Coupé et al., 2020). ROIs included for regional SUV quantification were the frontal, occipital, and temporal whole‐cortex ROIs, and further granular segmentation of the accumbens, amygdala, basal forebrain, brainstem, caudate, cerebellum (used as reference), cingulate, entorhinal, hippocampus, insula, pallidum, parahippocampus, putamen, thalamus, ventral DC, ventricle, and cortical white matter. Given the spatial resolution of PET imaging overall, the use of larger, more generalized ROIs is better‐suited for quantification of PET uptake/binding. Reconstructed PET full width at half maximum (FWHM) image resolution is ~4 mm isotropic, and many of the granular parcellations composing the entire AssemblyNet ROIs are in the order of a few cm^3^, increasing probability of spillover and partial volume effects that may lead to the inaccurate quantification of these smaller ROIs. Furthermore, since this was a proof‐of‐concept study, in the absence of pre‐existing data, we first hypothesized microglial activation would be dispersed across the whole brain given the systemic nature of cancer survivors' symptomatology. Thus, any choice of specific granular regions (outlined above) was based on elevated binding evident across other neurological diseases (discussed further in Section 4). A whole‐brain analysis was also conducted. SUV maps were generated by normalizing voxel‐wise SUV to the cerebellum, a previously published pseudo‐reference region (Lyoo et al., 2015). TSPO PET studies examining mild cognitive impairment, stroke, and Alzheimer's disease patients have used the cerebellum as reference because this region has shown to be relatively unaffected by disease pathology such as neuroinflammation, and spared the effects of brain lesion, diaschisis and/or neurodegeneration (Braak & Braak, 1991; Gerhard et al., 2005; Gulyas et al., 2012; Lyoo et al., 2015; Mattiace et al., 1990; Morris et al., 2018; Price et al., 2006; Wood, 2003), making it a clinically meaningful reference region for this type of study. Many studies with [^11^C]‐PBR28 utilize dynamic scans and carry out kinetic modeling using an input function derived from collection of serial arterial blood samples. Arterial cannulation is invasive and complicates measurement. Kinetic modeling with arterial blood sampling generally leads to high variability because of the difficulty of the method. Several studies have shown that simpler non‐invasive approaches utilizing SUV or SUVR may be sensitive to changes in TSPO levels (Lyoo et al., 2015). In the proposed study, we found no significant difference in TSPO genotype between the two groups investigated (cancer survivors vs. matched healthy controls), confirming the rigor of our findings using SUVR.

### 2.7 Peripheral measures of inflammation: blood serum cytokine markers

Blood samples for the protein (TSPO genotype), and cytokine assays were collected by a Registered Nurse Practitioner (RNP) from The Vanderbilt Hormone Assay & Analytical Services Core. 10 IU heparin per ml of blood were collected in a EDTA coated tube and centrifuged for ~10‐min at 4°C. The sample was then aliquoted into a Sarstedt tube #55.526 with push cap, and appropriately labeled with date, sample ID, research ID, and assay type. Samples were then stored at −20 to −80°C at the Vanderbilt Hormone Assay & Analytical Services Core for assay analysis.

### 2.8 Assay analysis

Assays (CRP, GM‐CSF, IFN‐γ, IL‐1β, IL‐4, IL‐6, IL‐10, IL‐12(p40), IL‐12(p70), TNF‐α, TGF‐β1, TGF‐β2, TGF‐β3) were analyzed using standardized protocols from the Luminex MILLIPLEX map human cytokine/chemokine magnetic bead panel—premixed 29 plex—immunology multiplex x‐map technology via the MagPix system (HCYTOMAG‐60K and TGFBMAG‐64K‐03). Reactants of the assays were attached to the surfaces of tiny, fluorescent microspheres. Each set of microspheres carried a unique biological reagent distinguishable by internal dye ratios. Identification of the analyte was based upon specific fluorescent emission spectra of the bead associated with the analyte. Two LEDs with high‐speed digital signal processors and computer algorithms then distinguished which analyte was carried on each microsphere while quantifying the reaction based on fluorescent reporter signals. This instrumentation allowed for the analysis of multiple analytes from a single aliquot of sample.

### 2.9 Clinical outcomes

The following self‐report surveys were collected from all participants:

*Vanderbilt Head and Neck Symptom Survey (VHNSS) version 2.0 plus general symptom survey (GSS)* is a validated tool to measure symptom burden and functional deficits in head/neck cancer and its treatment (Cooperstein et al., 2012).

*Neurotoxicity Rating Scale (NRS)* is a self‐report 37 item tool examining neurocognitive symptoms associated with neurotoxicity of medical treatment (Aldenkamp et al., 1995).

*PROMIS‐29* assesses seven domains (depression, anxiety, physical function, pain interference, fatigue, sleep disturbance, participation in social roles/activities) using 5‐point Likert scale, across 29‐items. It demonstrates excellent internal consistency and ability to compare across conditions and with normalized data (Katz et al., 2017).

*Behavior Rating Inventory of Executive Function—Adult version (BRIEF‐A)* is a standardized 75‐item measure that captures views of an adult's executive functions or self‐regulation in his or her everyday environment. It measures nine non‐overlapping theoretically and empirically derived clinical domains: inhibit, self‐monitor, plan/organize, shift, initiate, task monitor, emotional control, working memory, and organization of materials (Roth et al., 2005).

### 2.10 Overall statistical analysis

This study was proof‐of‐concept and the first investigation into microglial activation in cancer survivors presenting chronic systemic symptoms versus healthy controls. Thus, we had limited data available to conduct power analysis, and did not want to recruit an unnecessarily large sample considering the high level of symptom burden in the studied clinical cohort. As such, to test the difference of SUVR between cancer survivors and healthy controls, with 10 participants per group, we could detect a relative mean difference (to standard division) of 1.3 with 80% power and two‐sided type‐I error rate of 0.05. For example, if the standard deviation is 0.1, the mean difference that our data would be able to detect is 0.13. Prior to performing the primary analyses, descriptive statistics and graphical summaries were obtained for the PET‐MR data, MRI/DTI, serum cytokine, and clinical assessment outcomes to check for outliers, missing data, and the need for transformations or non‐parametric methods. It was then determined if there were any statistical significance in demographic profile (i.e., age, sex, years of education, and so on) or TSPO genotype between groups (cancer survivors vs. healthy controls) to ensure rigorous matching. One‐way ANOVA compared differences in age and education between the two groups (cancer survivors vs. matched healthy controls), and to check no significant differences in categorical demographic data (such as sex and race) between groups, the chi‐square co‐efficient was used. Clinical survey data was analyzed using Independent Samples *t*‐tests. Repeated measures mixed model analysis with Bonferroni correction for multiple comparisons, were conducted separately to test main effects (Region—see Section 2.6, and Hemisphere) and interactions in mean PET ([^11^C]‐PBR SUVR) data values, with between group factor (non‐CNS cancer survivors vs. matched healthy controls). Where assumption of sphericity was violated, Greenhouse Geisser correction was applied. Partial eta squared (*η*_p_^2^) effect sizes were also calculated and reported as useful information for future studies, where 0.01 observes a small, 0.06 medium, and >0.14 large, effect. Significant main effects/interactions were then unpacked further with Independent Samples *t*‐tests, and Cohen's *d* independent samples effect sizes were reported for future studies, where *d* = 0.2 is a small, *d* = 0.05 medium, and *d* = 0.08 large, effect. One‐way ANOVA was applied to test group differences in DTI (FA) data values, MRI cortical thickness, and blood serum cytokine markers. Post‐hoc interactions between the various data types (PET, DTI, blood serum, clinical outcomes) were examined using linear association Pearson and point‐biserial correlation, split by Group, to cross‐correlate continuous‐continuous and categorical‐continuous datasets. Because correlation coefficients are effects sizes, correction was not applied to p values to account for multiple comparisons, rather the adjusted *R* squared (*R* ^2^_adj_) was calculated and reported as percentage.

## 3 RESULTS

There were no significant differences in age (*p* = .13), sex (*p* = .29), years of education (*p* = .18), ethnicity (*p* = .86), and/or TSPO genotype (*p* = .34), between the cancer survivor and matched healthy control groups.

### 3.1 Brain tissue microstructural integrity: diffusion tensor imaging fractional *anisotropy*

Generally, no significant findings were evident between groups for any ROI examined in mean fractional anisotropy. Except, in the left hemisphere superior cerebellar peduncle *F*(1, 18) = 5.304, *p* = .033 (*η*_p_^2^ = 0.23), cancer survivors showed significantly lower mean fractional anisotropy compared to matched health controls; 0.493 (0.045) versus 0.538 (0.042).

### 3.2 MRI T1‐weighted cortical thickness

To note we conducted this analysis posthoc to further investigate MRI‐based measures of CNS integrity. Of the 32 ROIs explored, only two regions showed significance, but in opposing pattern of results; (1) cancer survivors showed significantly higher left inferior‐temporal cortical thickness *F*(1, 18) = 5.344, *p* = .033 (*η*_p_^2^ = 0.00^i^); and (2) the opposite direction was observed for right hemisphere precentral area, with cancer survivors showing significant cortical thinning compared with healthy controls *F*(1, 18) = 4.933, *p* = .039 (*η*_p_^2^ = 0.22). Table 5 provides an overview of MRI T‐1 weighted cortical thickness values in cancer survivors versus matched healthy controls.

### 3.3 Microglial activation (neuroinflammation): [ ^11^C]‐PBR28 SUVR

A main effect of Region *F*(22, 369) = 40.185, *p* < .001, *ƞ*_p_^2^ = 0.691, and Region × Group interaction *F*(22, 396) = 2.113, *p* = .003, revealed cancer survivors showed significantly elevated [^11^C]‐PBR28 binding in the following brain ROIs: caudate *t*(18) = −2.187, *p* = .042 (*d* = 0.056); occipital *t*(18) = −2.605, *p* = .018 (*d* = 0.050); and temporal *t*(18) = −2.528, *p* = .025 (*d* = 0.031), regions. Cancer survivors also showed significantly elevated [^11^C]‐PBR28 binding in the fourth ventricle *t*(18) =2.328, *p* = .032 (*d* = 0.083). Specific SUVR values are presented in Tables 3 and 4.

**TABLE 3:** Mean [^11^C]‐PBR28 standardized uptake value ratio (SUVR) by region of interest (ROI).

	Cancer survivors	Healthy controls	Comparison
Whole brain	.905 (.030)	.890 (.045)	*p* = .389	
Accumbens	.944 (.125)	.957 (.045)	*p* = .759	
Amygdala	.981 (.122)	1.001 (.076)	*p* = .659	
Basal forebrain	1.019 (.114)	1.024 (.057)	*p* = .913	
Brainstem	1.083 (.185)	1.153 (.072)	*p* = .277	
Caudate	**.771 (.055)**	**.715 (.058)**	*p* = .**042**	
Cingulate	.965 (.046)	.968 (.041)	*p* = .874	
Entorhinal	1.015 (.058)	.956 (.090)	*p* = .0.98	
Frontal	.906 (.031)	.908 (.066)	*p* = .908	
Hippocampus	.984 (.082)	.985 (.053)	*p* = .951	
Insula	.958 (.068)	.966 (.037)	*p* = .733	
Occipital	**1.005 (.051)**	**.946 (.049)**	*p* = .**018**	
Pallidum	.947 (.132)	.957 (.057)	*p* = .836	
Parahippocampus	.965 (.040)	.926 (.064)	*p* = .116	
Putamen	.950 (.081)	.924 (.057)	*p* = .429	
Temporal	**.973 (.020)**	**.939 (.039)**	*p* = .**021**	
Thalamus	1.021 (.125)	1.067 (.037)	*p* = .285	
Ventral DC	.986 (.159)	1.047 (.075)	*p* = .283	
Lateral ventricle	.667 (.122)	.620 (.151)	*p* = .451	
Whitematter	.842 (.052)	.832 (.052)	*p* = .667	
CSF third ventricle	.934 (.123)	1.031 (.160)	*p* = .148	
CSF fourth ventricle	**.893 (.071)**	**.980 (.094)**	*p* = .**032**	

*Note*: Values in bold font highlights statistically significant findings.

**TABLE 4:** Mean [^11^C]‐PBR28 standardized uptake value ratio (SUVR) by brain lateralization.

	Cancer survivors		Healthy controls		Between group comparison		
	L	R	L	R	L		R
Accumbens	.948 (.120)	.940 (.132)	.963 (.035)	.949 (.060)	*p* = .711		*p* = .841
Amygdala	.987 (.126)	.975 (.123)	1.004 (.071)	1.000 (.084)	*p* = .710		*p* = .624
Basal forebrain	1.022 (.113)	1.017 (.117)	1.023 (.064)	1.025 (.055)	*p* = .973		*p* = .852
Caudate	.762 (.053)	**.779 (.059)**	.719 (.059)	**.713 (.060)**	*p* = .100		*p* = .**022**
Cingulate	.964 (.051)	.966 (.044)	.966 (.041)	.971 (.042)	*p* = .941		*p* = .793
Entorhinal	.990 (.066)	1.038 (.062)	.943 (.082)	.969 (.101)	*p* = .173		*p* = .084
Frontal	.908 (.031)	.903 (.033)	.903 (.065)	.913 (.068)	*p* = .833		*p* = .678
Hippocampus	.995 (.101)	.972 (.069)	.990 (.055)	.981 (.055)	*p* = .883		*p* = .757
Insula	.959 (.066)	.956 (.072)	.963 (.041)	.970 (.034)	*p* = .901		*p* = .582
Occipital	**1.005 (.058)**	**1.004 (.048)**	**.941 (.047)**	**.950 (.052)**	*p* = .**015**		*p* = .**026**
Pallidum	.948 (.120)	.946 (.147)	.963 (.051)	.950 (.066)	*p* = .727		*p* = .937
Parahippocampus	.958 (.043)	.972 (.041)	.919 (.057)	.932 (.073)	*p* = .102		*p* = .148
Putamen	.948 (.076)	.952 (.087)	.921 (.056)	.928 (.060)	*p* = .377		*p* = .484
Temporal	**.973 (.024)**	***.973 (.018)***	**.930 (.037)**	***.947 (.042)***	*p* = .**006**		*p* = . ***088***
Thalamus	1.016 (.129)	1.021 (.122)	1.059 (.039)	1.074 (.037)	*p* = .326		*p* = .252
Ventral DC	.985 (.158)	.986 (.160)	1.047 (.073)	1.048 (.078)	*p* = .278		*p* = .290
Whitematter	.843 (.052)	.842 (.051)	.830 (.053)	.834 (.052)	*p* = .590		*p* = .751
Ventricular CSF	.643 (.133)	.645 (.132)	.586 (.154)	.588 (.143)	*p* = .390		*p* = .364

*Note*: Values in bold font highlights statistically significant findings.

**TABLE 5:** Mean MRI T‐1 weighted cortical thickness.

	Cancer survivors		Healthy controls		Between group comparison	
	L	R	L	R	L	R
Mean thickness	2.44 (.09)	2.46 (.10)	2.45 (.09)	2.45 (.09)	*p =* .912	*p =* .907
Caudal anterior cingulate	2.55 (.11)	2.54 (.21)	2.56 (.23)	2.41 (.25)	*p* = .897	*p =* .230
Caudal middle frontal	2.55 (.19)	2.48 (.15)	2.60 (.13)	2.57 (.15)	*p =* .574	*p =* .184
Cuneus	1.83 (.07)	1.83 (.08)	1.88 (.18)	1.88 (.18)	*p* = .432	*p =* .402
Entorhinal	3.31 (.19)	3.27 (.31)	3.18 (.26)	3.16 (.37)	*p* = .205	*p =* .491
Fusiform	2.58 (.09)	2.68 (.12)	2.62 (.14)	2.63 (.14)	*p* = .454	*p =* .381
**Inferior parietal**	2.43 (.12)	2.42 (.12)	2.43 (.13)	2.40 (.13)	*p* = .931	*p =* .843
Inferior temporal	**2.70 (.08)**	2.77 (.09)	**2.61 (.10)**	2.72 (.06)	***p =* .033**	*p =* .198
Isthmus cingulate	2.44 (.13)	2.48 (.18)	2.38 (.14)	2.43 (.18)	*p* = .283	*p =* .494
Lateral occipital	2.13 (.12)	2.15 (.11)	2.11 (.11)	2.14 (.09)	*p =* .734	*p =* .863
Lateral orbitofrontal	2.68 (.17)	2.72 (.11)	2.64 (.11)	2.65 (.14)	*p =* .560	*p =* .212
Lingual	1.87 (.13)	1.98 (.16)	1.94 (.14)	1.99 (.17)	*p* = .234	*p =* .968
Medial orbitofrontal	2.54 (.10)	2.53 (.11)	2.58 (.16)	2.50 (.21)	*p* = .589	*p =* .749
Middle temporal	2.64 (.10)	2.76 (.11)	2.70 (.15)	2.70 (.14)	*p* = .310	*p =* .249
Parahippocampus	2.63 (.25)	2.77 (.26)	2.60 (.21)	2.61 (.12)	*p* = .776	*p =* .089
Paracentral	2.30 (.16)	2.36 (.22)	2.31 (.12)	2.45 (.14)	*p* = .877	*p =* .276
Parsopercularis	2.58 (.17)	2.58 (.18)	2.61 (.12)	2.62 (.15)	*p* = .651	*p =* .598
Parsorbitalis	2.69 (.10)	2.74 (.20)	2.65 (.09)	2.62 (.15)	*p =* .272	*p =* .146
Parstriangularis	2.44 (.15)	2.45 (.10)	2.42 (.17)	2.41 (.23)	*p =* .708	*p =* .664
Pericalcarine	1.49 (.12)	1.48 (.08)	1.46 (.14)	1.55 (.14)	*p =* .631	*p =* .221
Postcentral	2.07 (.14)	2.13 (.21)	2.05 (.11)	2.05 (.11)	*p =* .750	*p =* .315
Posterior cingulate	2.44 (.14)	2.50 (.15)	2.39 (.12)	2.51 (.16)	*p =* .402	*p =* .927
**Precentral**	2.57 (.16)	**2.34 (.29)**	2.60 (.13)	**2.57 (.13)**	*p =* .563	***p =* .039**
Precuneus	2.34 (.17)	2.39 (.13)	2.37 (.17)	2.37 (.13)	*p =* .646	*p =* .739
Rostral anterior cingulate	2.84 (.17)	2.71 (.18)	2.75 (.20)	2.60 (.10)	*p =* .264	*p =* .113
Rostral middle frontal	2.45 (.13)	2.45 (.08)	2.46 (.18)	2.42 (.16)	*p =* .903	*p =* .621
Superior frontal	2.69 (.18)	2.65 (.20)	2.71 (.15)	2.72 (.15)	*p =* .746	*p =* .397
Superior parietal	2.18 (.13)	2.10 (.14)	2.18 (.13)	2.11 (.12)	*p =* .924	*p =* .872
Superior temporal	2.71 (.08)	2.84 (.12)	2.72 (.12)	2.80 (.18)	*p =* .811	*p =* .570
Supramarginal	2.53 (.13)	2.52 (.17)	2.50 (.14)	2.49 (.17)	*p =* .643	*p =* .722
Transverse temporal	2.25 (.17)	2.48 (.29)	2.31 (.24)	2.41 (.30)	*p =* .550	*p =* .601
Insula	3.16 (.14)	3.05 (.20)	3.10 (.12)	3.00 (.14)	*p =* .377	*p =* .530

*Note*: Values in bold font highlights statistically significant findings.

**TABLE 6:** Overview of main findings/interactions across the discrete study measures.

	Main findings in non‐CNS cancer survivors compared to healthy controls	Summary of interactions in non‐CNS cancer survivors between main findings		
		FA superior cerebellar peduncle	Chronic symptomatology (VHNSS, GSS, neurotoxicity)	BRIEF neurocognitive functioning (↑ BRIEF scores = ↑ impairment)
Centralized inflammation caudate	**↑**	X	X	X
Centralized inflammation temporal	**↑**	X	+ Corr w/: VHNSS; neurotoxicity	X
Centralized inflammation occipital	**↑**	X	X	− Corr w/: emotion control
Centralized inflammation CSF 3rd ventricle	X	X	X	X
Centralized inflammation CSF 4th ventricle	**↓**	X	+ Corr w/: sleep disturbance	+ Corr w/: shift
Peripheral pro‐inflammatory cytokines	X	X		
Peripheral anti‐inflammatory cytokines	X	X		
Peripheral blood C‐Reactive protein	X	−Corr (↓FA = ↑CRP)		
White matter FA superior cerebellar peduncle	**↓**		−Corr w/: VHNSS; GSS; neurotoxicity	X
White matter FA all other ROIs	X			
MRI right hemisphere precentral cortical thickness	↓	X	X	X

*Note*: centralized inflammation = [^11^C]‐PBR28 SUVR data; peripheral cytokines = blood serum assay data.

Abbreviations: CSF, cerebrospinal fluid; FA, fractional anisotropy from DTI data; GSS, VHNSS General Symptom Survey; VHNSS, Vanderbilt Head and Neck Symptom Survey Version 2.0 total score.

When stratifying [^11^C]‐PBR28 binding by lateralization, a main effect of Region *F*(17, 306) = 50.016, *p* < .001, *ƞ*_p_^2^ = 0.735, and Region × Hemisphere interaction *F*(17, 306) = 3.799, *p* < .001, *ƞ*_p_^2^ = 0.174, showed that cancer survivors yielded significantly higher values compared to healthy controls in the caudate for the right hemisphere *t*(18) = −2.502, *p* = .022 (*d* = 0.059), that was not observed in the left hemisphere *p* = .100. Furthermore, significantly higher binding values were observed in the left *t*(18) = −2.687, *p* = .015 (*d* = 0.053) and right *t*(18) = −2.432, *p* = .026 (*d* = 0.050), occipital hemispheres in cancer survivors compared with healthy controls. This was also observed with the left temporal hemisphere *t*(18) = −3.131, *p* = .006 (*d* = 0.031), that was not evident in right hemisphere *p* = .096.

**FIGURE 2:**
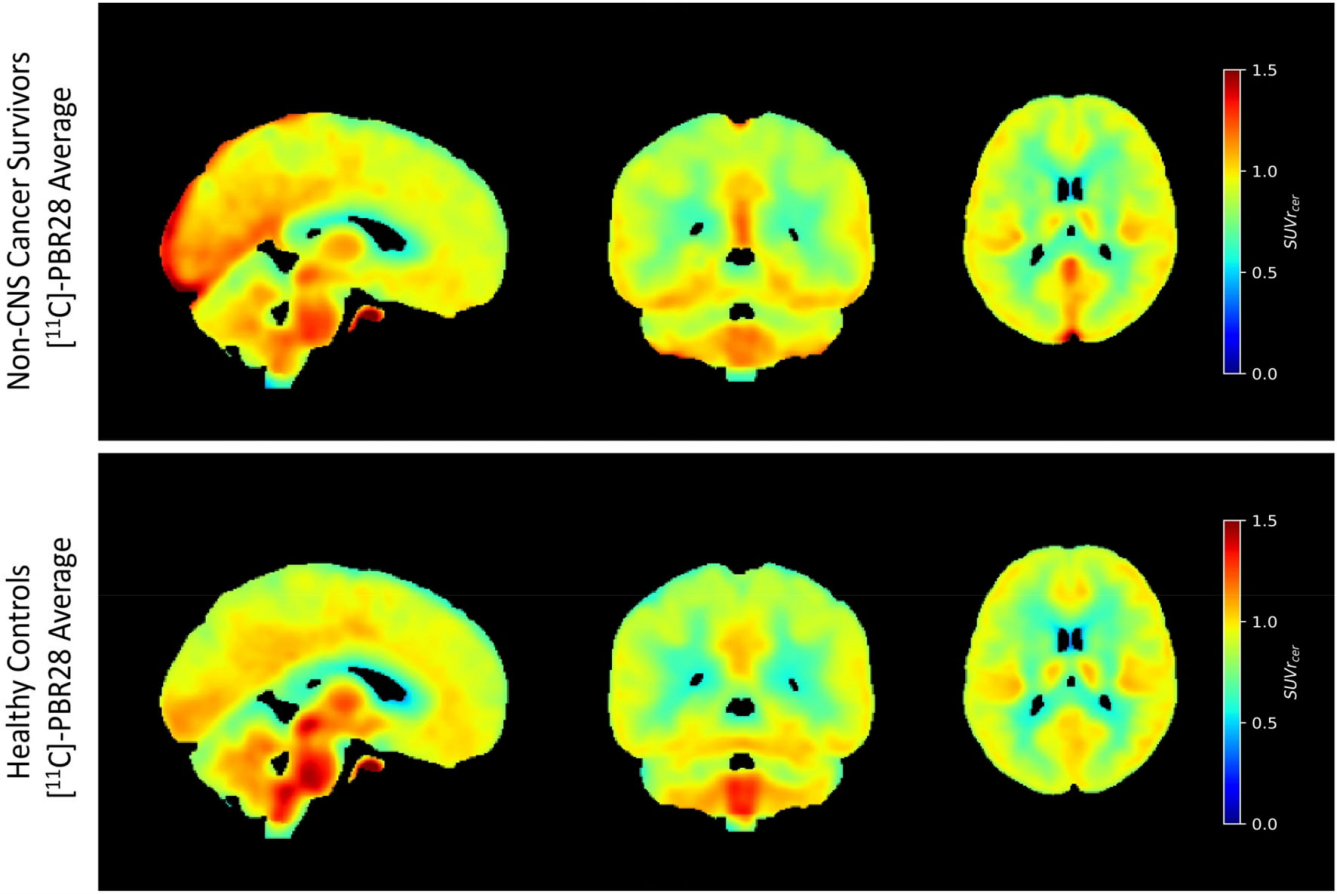
Average [^11^C]‐PBR28 standardized uptake value ratio (SUVR) maps registered to AssemblyNet standard space for survivors of head and neck cancers (*n* = 10) versus matched healthy controls (*n* = 10) are displayed in orthogonal views. The whole cerebellum was used as a reference region for SUVR calculation according to previous studies and standardized protocols. Cancer survivors have greater uptake of TSPO tracer [^11^C]‐PBR28 in posterior and medial regions compared to matched healthy controls.

Visualization of average [^11^C]‐PBR28 SUVR in non‐CNS cancer survivors versus matched healthy controls are displayed in orthogonal views in Figure 2. There was no region with significantly elevated [^11^C]‐PBR28 binding in matched controls compared with cancer survivors.

### 3.4 Peripheral inflammation: blood serum cytokine assays

There were no significant differences between the cancer survivors and matched healthy controls in any of the peripheral inflammatory cytokine biomarkers: blood serum CRP, CSF, IFN‐γ, IL‐10, IL‐12(p40), IL‐12(p70), IL‐1β, IL‐4, IL‐6, IL‐8, TNF‐α, TGF‐β1,2, or 3.

### 3.5 Correlations between [ ^11^C]‐PBR28 SUVR and clinical outcomes

In the cancer survivors, increased [^11^C]‐PBR28 SUVR in occipital region correlated with decreased emotion control on the Behavior Rating Inventory of Executive Function/BRIEF (*r* = −0.664, *p* = .036, *R* ^2^ adj = 34.16). No correlations were evident in the matched healthy control group.

For temporal region, increased [^11^C]‐PBR28 SUVR correlated with greater symptomatic dysfunction/severity on the Vanderbilt Head and Neck Symptom Survey Version 2.0 assessment (*r* = 0.850, *p* = .002, *R* ^2^ adj = 68.78), and higher neurotoxicity scores (*r* = .680, *p* = .031, R^2^ adj =39.52). No correlations were evident in the matched healthy control group.

### 3.6 Post‐hoc correlations: [ ^11^C]‐PBR28 SUVR and fractional anisotropy

Since there was a significant difference in white matter fiber tract integrity of the superior cerebellar peduncle (SCP) between cancer survivors and matched healthy controls, we examined whether this finding correlated to microglial activation. Firstly, no significant findings were evidence in the matched healthy control group. For cancer survivors, reduced fractional anisotropy in both left and right hemispheres correlated with increased [^11^C]‐PBR28 SUVR for the whole brain/wb (right SCP: *r* = −0.642, *p* = .045, *R* ^2^ adj = 33.87); amygdala (left SCP: *r* = −0.710, *p* = .022, *R* ^2^ adj = 44.21), right SCP: *r* = −0.727, *p* = .017, *R* ^2^ adj = 46.96); basal forebrain (right SCP: *r* = −0.640, *p* = .046, *R* ^2^ adj = 33.58); brainstem (right SCP: *r* = −0.690, *p* = .027, *R* ^2^ adj = 41.06); hippocampus (left SCP: *r* = −0.718, *p* = .019, *R* ^2^ adj = 45.50, right SCP: *r* = −0.745, *p* = .013, *R* ^2^ adj = 49.94); whitematter (right SCP: *r* = −0.642, *p* = .046, *R* ^2^ adj = 33.87).

### 3.7 Post‐hoc correlations: fractional anisotropy and clinical outcomes

Exclusive to cancer survivors, decreased fractional anisotropy of the left superior cerebellar peduncle correlated with higher values on the Vanderbilt Head and Neck Symptom Survey/VHNSS total score (*r* = −0.667, *p* = .035, *R* ^2^ adj = 37.55). Reduced fractional anisotropy of the right superior cerebellar peduncle correlated with higher scores on the VHNSS General Symptom Survey/GSS (*r* = −0.691, *p* = .027, *R* ^2^ adj = 41.22), and neurotoxicity (*r* = −0.707, *p* = .022, *R* ^2^ adj = 43.73). No significant findings were evident for any of the matched healthy control data.

### 3.8 Post‐hoc correlations: [ ^11^C]‐PBR28 SUVR and MRI cortical thickness

There were no significant findings in either group.

Table 6 provides an overview matrix of the main findings/interactions across all the discrete study measures.

## 4 DISCUSSION

We present new data that (1) shows microglial activation, a molecular marker of neuroinflammation, in a cohort of late non‐central nervous system/CNS cancer survivors; (2) shows the same patient group with intact white matter microstructural integrity, a neuronal marker fiber tract organization, where cortical white matter tracts have also been shown to be sensitive to underlying neurodegenerative disease pathology (Young et al., 2020); and (3) for the first time links the presentation of chronic systemic symptoms with significant microglial activation. Head and neck cancers are non‐CNS cancers, therefore data indicating inflammation of the central nervous system indexed by microglia activation is significant given that the cancer and its treatments terminated in this cohort an average of ~4.6 years previously (range from 1 to 16 years). Furthermore, our cohort included survivors exposed to a range of multimodal cancer treatments (including surgery + ChemoXRT, induction + surgery + ChemoXRT, induction + ChemoXRT, ChemoXRT only, and radiotherapy only), with an average length of 6.58 weeks radiation treatment, average number of 5.83 Induction Chemotherapy cycles, and average number of 7.22 ChemoXRT cycles, for those patients exposed to whichever treatment program.

### 4.1 Centralized versus peripheral inflammation

Another important finding of this study pertains to the peripheral cytokine markers of inflammation, namely the lack of significant difference between survivors and healthy controls in these measures. Microglia are fundamental neuroimmune cells of the central nervous system providing a first‐line neuroprotective mechanism for acute insult to the brain, in addition to their involvement in chronic pathological processes such as inflammation, stroke, viral/bacterial infections, and neurodegenerative decline (Augusto‐Oliveira et al., 2019; Gómez‐Nicola et al., 2013; Hernandez‐Ontiveros et al., 2013; Jiang et al., 2020; Li et al., 2021; Lull & Block, 2010; Woodburn et al., 2021; Yin et al., 2017). Complete understanding of microglia's mechanistic role/s remains a developing field. Simplified frameworks propose that activated microglia can acquire different phenotypes (M1 and M2) depending on cues in the surrounding microenvironment. The M1 phenotype is the “classic” first responder to toxin/injury/insult releasing pro‐inflammatory cytokines and neurotoxic molecules that promote inflammation and cytotoxic reactions. M2 microglia (and sub‐components a–c, *M2d is examined primarily in tumor‐associated macrophages and its significance in microglia is presently unclear (Wendimu & Hooks, 2022)) secrete anti‐inflammatory cytokines and nutrient factors that promote the function of repair, regeneration, ultimately restoring homeostasis (Orihuela et al., 2016; Tu et al., 2021). Peripheral cytokines both activate and are secreted by microglia phenotypes. These cytokines can also be broadly classified as pro‐ versus anti‐inflammatory in function, albeit some are dual‐purposed. Pro‐inflammatory cytokines include IL‐1B, IL‐6,^ii^ IL‐8, IL‐12, TNF‐α, and interferons (i.e., IFN‐γ), facilitating inflammatory reactions and stimulating immunocompetent cells. Anti‐inflammatory cytokines include IL‐4, IL‐6, IL‐10, IL‐11, IL‐13, TGF‐β, inhibiting inflammation and suppressing immune cells (Liu et al., 2021). This study included both pro‐inflammatory (IL‐1B, IL‐6, IL‐8, IL‐12 (p40, p70), TNF‐α, and IFN‐γ), and anti‐inflammatory (IL‐4, IL‐6, IL‐10, TGF‐β), peripheral cytokine blood serum markers with no significant findings evident between cancer survivors and matched controls. The lack of statistical difference between groups in any of the peripheral inflammatory cytokine markers we collected is informative considering the significant microglial activation (centralized inflammation) findings. One explanation might be that since survivors were so far out from cancer remission and cancer treatment completion (average ~ 4.6 years, range 1–16 years), peripheral inflammation resolved and/or was no longer detectable while centralized neuroimmune toxic response continued, causing chronic systemic symptomatology. This is salient because it would also support a peripheral‐to‐centralized neuroimmune framework, where levels of peripheral inflammatory cells and mediators (i.e., as a consequence of the cancer and/or its treatments) become unmanageable and progress to toxification of the entire CNS, or centralized neuroinflammation (Schoenberg & Gonzalez, 2023). When one component of the inflammatory chain becomes dysregulated, continued inflammatory response ensues in the absence of the original peripheral cause (cancer/its treatments) which is no longer observable, say through standardized follow‐up bloodwork. This peripheral‐to‐central neuroinflammatory process is multicellular and mediated by neuroglial cells of the CNS. Chronic neuroglial activation of the CNS (neuroinflammation) presents in neuronal dysfunction and injury across diverse clinical populations (Bachiller et al., 2018). Future large scale longitudinal studies will be able to develop this hypothesis by including multiple patients across stage of illness and survivorship to concretely ascertain whether those patients whose peripheral inflammation does not extend to toxification of the CNS do not present chronic systemic symptomatology, which we think is highlight likely to be the case. Related to this, a further explanation is that activated microglia do not solely secrete the cytokines we examined; chemokines, nitric oxide, and reactive oxygen species may also be potentially released peripherally once microglia are activated with varying degrees of benefit/harm to the surrounding microenvironment (Harry, 2013). Moreover, microglia release neurotransmitters and other cell communicators that affect synaptic activity both directly, and by extracellular processes including cell‐released exosomes and microvesicles containing DNA, RNA, proteins and lipids which facilitate transfer from glia to neurons, for example (Augusto‐Oliveira et al., 2019). Because we did not collect all possible peripheral measures associated with neuroimmune response, we cannot piece together the full picture at this juncture. However, the data gathered here makes the connection between microglia activation and clinical presentation, supporting the significance of activated microglia (centralized inflammation) and manifestation of chronic systemic symptomatology in late non‐CNS cancer survivors. We also found significant differences in electrophysiological event‐related potential (ERP) data that are peripheral measures of neurotransmission between our non‐CNS cancer survivor cohort and matched healthy controls, which we report elsewhere. Centralized measures of inflammation are currently lacking in clinical practice to correctly recognize systemic symptoms in non‐CNS cancer survivors. Common follow‐up cancer survivor care involves bloodwork that based on the present findings might not represent the right tools to assess chronic systemic symptomatology and/or long‐term CNS complications.

### 4.2 Genetic and transcription considerations

One potential future direction of investigation for the question as to why some cancer survivors go on to develop detrimental levels of microglial activation while others not; may be addressed by relatively recent evidence pointing to a subset of disease‐associated microglia (or DAM) that have specialized transcriptional function and are linked to genes connected with neurodegenerative conditions (Deczkowska et al., 2018; Keren‐Shaul & Spinrad, 2017). For example, the TREM2 gene is expressed exclusively by disease‐associated microglia/DAM in the brain and is modulated by inflammation (Gratuze et al., 2018). The TREM2 receptor has been connected to various pathophysiology in brain diseases such as dementia (Carmona et al., 2018; Edwin & Henjum, 2020; Gratuze et al., 2018; Ulland & Colonna, 2018; Wang et al., 2020; Zhao et al., 2022), and TREM2 signaling is involved in the regulation of critical microglial functions encompassing proliferation, cytokine release, survival and metabolism (Zhao et al., 2022). Animal models and to lesser degree clinical evidence in humans at present, support the hypothesis that disease‐associated microglia/DAM entail specialized sensory mechanism/s and signaling pathways towards the detection of neural tissue damage, via neurodegeneration‐associated molecular patterns (NAMPs); a centralized framework developed from the peripheral immune system's pathogen‐ and damage‐associated inflammatory pattern detections (PAMPs and DAMPs, alluded to previously) (Deczkowska et al., 2018). Further investigation into the role of disease‐associated microglia/DAM, and interaction with genes (TREM2) and pathogenic signaling, may provide important information to predict which non‐CNS cancer patients will go on to develop detrimental (opposed to beneficial (Pons & Rivest, 2020) microglial activation and related systemic symptomatology post treatment/remission. The pragmatic implications of this concept lend to the prophylactic modification of disease‐associated microglia/DAM activity prior to cancer treatments in order to prevent the accumulation of CNS damage/toxification (Aldenkamp et al., 1995; McDade & Bateman, 2017), and centralized neuroinflammation in the first instance. For example, early work in neurodegenerative diseases supports an innovative therapeutic approach of targeting microglia‐specific inhibitory “checkpoints” to induce preventative disease‐associated microglia/DAM activation prior to manifestation of symptomatology (Keren‐Shaul & Spinrad, 2017). Theoretically this could be compelling for non‐CNS cancer patients as concurrent preventative measures prior to the onset of their cancer treatment program/s if predictive trajectories are established. Such framework/s could also be applied as intervention targets where prevention proves difficult because of complex pathobiological factors/interactions, particularly in switching microglial polarization (M1, M2), from a pro‐inflammatory to anti‐inflammatory phenotype once CNS toxification/damage has initiated.

### 4.3 Patterns of localization in microglial activation, functional anatomy, and clinical phenotypes

The proof‐of‐concept nature of this study meant we had minimal data to generate hypotheses in terms of which regions of interest would display microglial activation in non‐CNS cancer survivors. Based on the systemic presentation of symptomatology, we thus predicted dispersed microglial activation across the whole brain in our head and neck cohort from the outset. However, this was not the case; significantly increased microglial activation was spatially defined to regions of the caudate, temporal and occipital lobes. Initial findings within the literature suggest that patterns of microglial activation may characterize discrete neurodegenerative disorders and neurological conditions. The early clinical stages of multiple system atrophy, an adult‐onset progressive brain disease, shows widespread microglial activation compared with healthy controls. Specifically, those with the Parkinsonian phenotype measure increased binding in the caudate nucleus, putamen, pallidum, precentral gyrus, orbitofrontal cortex, presubgenual anterior cingulate cortex, and the superior parietal gyrus (Kübler et al., 2019). However, these neuroinflammatory changes do not appear to correlate with specific clinical parameters, rather might provide a first indicator of early‐stage neurological decline. Alternatively, Huntington's disease, another brain disease that causes progressive neuronal cell death but unlike multiple system atrophy is closely linked with genetic factors, shows localized microglial activation. Increased microglial activation has been revealed in Huntington Disease gene carriers, that also correlate with disease stage (Pavese et al., 2006; Politis et al., 2008, 2011, 2015; Rocha et al., 2021; Tai et al., 2007). For example, increased striatal [^11^C]‐PK11195 binding has been reported to significantly correlate with disease severity (Pavese et al., 2006). Other regions affected by neuroinflammation/microglial activation in Huntington disease patients pertain to the prefrontal cortex and anterior cingulate. These brain areas are responsible for higher‐order cognitive executive functioning, processes that are also impaired as the disease progresses. The [^11^C]‐ER176 PET tracer has found increased microglial activation in Huntington disease patients in basal ganglia and hypothalamic structures such as putamen and pallidum (Rocha et al., 2021). These spatial patterns are also in line with Parkinson's disease patients showing significantly higher [^18^F]‐DPA714 binding compared to healthy controls bilaterally in frontal and basal ganglia contralateral regions suggesting nigro‐striatal‐frontal pathway of microglial activation dispersion in Parkinson's disease (Lavisse et al., 2021). Moreover, increased microglial activation appears to be associated with higher plasma cytokine levels in premanifest Huntington's disease gene carriers (Politis et al., 2015). In sum, although it may seem reasonable to first theorize comparisons between non‐CNS cancer survivor systemic symptomatology and neurodegenerative decline, the data presented here does not fully support this conjecture. While our non‐CNS cancer survivor data showed some striatal localization of microglia activation, such as the caudate; prefrontal regions were not affected in patients, nor did we observe any significant difference in plasma cytokine markers of peripheral inflammation between survivors and matched healthy controls.

Neurodegenerative disorders are complex, both in their clinical overlap and neuropathology. Topographical patterns of microglial activation in our non‐CNS cancer survivor cohort may connect more closely to early stages of Lewy body‐like dementia, which has distinct symptomatic clustering compared to progressed Alzheimer's and Parkinson's diseases that are linked to Lewy body pathology. For example, increased [^11^C]‐PK11195 TSPO binding has been observed in the caudate, frontal, temporal, and parietal cortices in cognitively mild forms of dementia with Lewy body patients, when compared with those presenting moderate cognitive impairment (Nacastro et al., 2020; Surendranathan et al., 2018). Microglial activation localized to the occipital region has been found in idiopathic rapid eye movement sleep disorder, a precursor to Lewy‐type α‐synucleinopathy (Stokholm et al., 2018). Increased microglial activation has been reported in temporal–parietal regions in the prodromal stages of Alzheimer's disease (Hamelin et al., 2016; Passamonti et al., 2018). Clusters of increased microglial activation have further been reported in temporal and occipital regions in asymptomatic and symptomatic SOD1 gene mutated amyotrophic lateral sclerosis carriers (Tondo et al., 2020). One discrepancy with our present findings is that microglial activation in these cases were also accompanied by peripheral inflammation when collected, such as increased IL‐2, IL‐7A, and IL‐8 (Surendranathan et al., 2018), but these peripheral markers did not correlate with centralized [^11^C]‐PK11195 TSPO binding. Other studies suggest that anterior temporal microglial activation (measured via [^11^C]‐PK11195 PET) might predict cognitive decline in patients with Alzheimer's disease pathology from mild cognitive impairment to early stage dementia (Malpetti et al., 2020). Our data contributes to this evidence base, although we are far from clear conclusions. Future investigation might include ascertaining if specific genetic or transcriptional data can predict cancer treatment trajectory to test whether those patients disposed to Lewy body pathology might develop increased microglial activation across treatment course, and thus develop systemic symptomatology. This hypothesis is supported by the established connection between increased risk of non‐CNS cancer patients developing long‐term cognitive impairments (Chen et al., 2015; van der Willik et al., 2018). Moreover, initial evidence suggests that cancer and dementia share genetic variants despite epidemiological patterns highlighting an inverse relationship between the two (Feng et al., 2017; van der Willik et al., 2018).

### 4.4 Limitations

First, this was a retrospective study, and so the design does not provide baseline data pre‐cancer and/or pre‐treatment as predictive trajectory measures. Such data could contribute towards answering why some non‐CNS cancer survivors go on to measure significant activated microglia and others not, which is an important piece of this puzzle moving forward. Second, the inclusion of genetic data for future studies could facilitate testing our aforementioned hypothesis that TREM2 signaling may enact disease‐associated microglia for detrimental/beneficial factors interacting with the manifestation of systemic symptomatology in a portion of non‐CNS cancer survivors. Third, we investigated microglial activation in a rather narrow cancer cohort, that is, head and neck survivors, that does not represent the full spectrum of non‐CNS cancers. This was due to pragmatic constraints since head and neck cancer was the only population available for recruitment and testing. However, chronic systemic symptomatology have been reported in a range of other non‐CNS cancer survivors, such as breast gynecological, prostate, and rectal/colon, that can continue for >10 years following treatment/cancer remission (Harrington, Hansen, et al., 2010). Findings need to be replicated across non‐CNS cancer types to robustly test the link between chronic systemic symptomatology and microglial activation (neuroinflammation). We suggest that a peripheral‐to‐central toxification from cancer and its treatments in any non‐CNS cancer will also yield a similar pattern of results, suggesting this proof‐of‐concept has applied scope. Fourth, PET SUVR has limitations since it does not use dynamic kinetic modeling. Many studies with [^11^C]‐PBR28 utilize dynamic scans and carry out kinetic modeling using an input function derived from collection of serial arterial blood samples. Arterial cannulation is invasive and complicates measurement. Several studies have shown that simpler non‐invasive approaches utilizing SUV or SUVR, what we used here, are still sensitive to changes in TSPO levels. Since we found no significant differences between groups in TSPO genotyping, this would further support the robustness of our SUVR results. Fifth, four cancer survivors were taking sedatives (likely to treat anxiety and/or poor sleep health), where preliminary evidence (Owen et al., 2014) has suggested the potential for sedatives to block TSPO uptake. This would suggest that TSPO uptake values could have theoretically been higher in these patients. Controlling for pharmacotherapy in future larger scale studies may be something to consider, if feasible and/or ethical to request patients to discontinue their medication regimes (that are treating their chronic systemic symptoms) prior to scanning. In this example, it appears the medications used would drive TSPO uptake in the opposite direction from what we found making our findings even more compelling. Finally, our study design renders limited information to disentangle whether the significant microglial activation in our cancer survivor cohort was the result of neuroimmune response to the cancer itself or the subsequent cancer treatments. A longitudinal design that follows patients across their trajectory from diagnosis to treatment completion would be necessary, and this is a planned next step for a larger scale study based on the findings from our “proof‐of‐concept” study.

### 4.5 Synthesis

We present new data that empirically supports a peripheral‐to‐centralized inflammatory response in non‐CNS cancer survivors, specifically those previously afflicted with head and neck cancer. Following resolution of the initial peripheral inflammation from the cancer/its treatments, in some cases injury/damage/toxification to the entire central nervous system occurs (despite the initial inflammatory response to cancer and/or its treatments in peripheral systems/structures), in turn manifesting chronic systemic symptoms evident years into remission. However, it is highly plausible that this process represents a form of neuroplasticity which can theoretically be reversed by anti‐neuroplastic interventions. For example, emerging evidence supports the significant cross‐correlation between the neuroimmune system and neuroplastic mechanisms in neuroimmune plasticity factors involved in central nervous system injury such as traumatic brain injury, spinal cord injury, and/or stroke (O'Reilly & Tom, 2020b; Tian et al., 2012), immune macroenvironment plasticity in cancer models (Allen et al., 2020), responsiveness to chronic pain (Pratscher et al., 2021; Sibille et al., 2016), and neuronal adaptation in mood disorders (Pittenger & Duman, 2007), to name a few. Incorporating measures to rule out centralized neuroinflammation and provide interventions that target such mechanisms without causing further toxification to the CNS (Schoenberg & Gonzalez, 2022, 2023), will have broad clinical impact for the non‐CNS survivor population.

## AUTHOR CONTRIBUTIONS

Conceptualization: Poppy L. A. Schoenberg, Barbara A. Murphy. Funding: Poppy L. A. Schoenberg, Barbara A. Murphy. Methodology: Poppy L. A. Schoenberg, Todd E. Peterson. Administration/Implementation: Poppy L. A. Schoenberg, Emily M. Mohr, Todd E. Peterson, Barbara A. Murphy. Supervision: Poppy L. A. Schoenberg. Analysis: Poppy L. A. Schoenberg, Alexander K. Song, Baxter P. Rogers. Visualization: Poppy L. A. Schoenberg, Alexander K. Song. Writing first draft: Poppy L. A. Schoenberg. Writing/editing: Poppy L. A. Schoenberg, Alexander K. Song, Baxter P. Rogers, Todd E. Peterson.

## FUNDING INFORMATION

This study was generously supported by the Ingram PSMP Complementary Medicine Endowment, the Vanderbilt Institute for Clinical and Translational Research (VICTR), and the Osher Center for Integrative Health at Vanderbilt. The Philips Vereos PET/CT scanner used in this study was supported by the National Institutes of Health (NIH S10OD012297).

## CONFLICT OF INTEREST STATEMENT

The authors declare no conflict of interest.

## Data Availability

The data that support the findings of this study are available from the corresponding author upon reasonable request.

## Endnotes

^i^
Although this effect size suggests a negligible, if any, real effect.

^ii^
IL‐6 serves both as pro‐ and/or anti‐inflammatory.
